# Effect of clinical factors on trajectory of functional performance in patients undergoing hemodialysis

**DOI:** 10.1080/0886022X.2020.1852090

**Published:** 2020-12-22

**Authors:** Jin-Bor Chen, Lung-Chih Li, Wen-Chin Lee, Sin- Hua Moi, Cheng-Hong Yang

**Affiliations:** a Division of Nephrology, Department of Internal Medicine, Kaohsiung Chang Gung Memorial Hospital and Chang Gung University College of Medicine, Kaohsiung, Taiwan; b Center of Cancer Program Development, E-Da Cancer Hospital, I-Shou University, Kaohsiung, Taiwan; c Department of Electronic Engineering, National Kaohsiung University of Science and Technology, Kaohsiung, Taiwan

**Keywords:** Age, albumin, hemodialysis, Karnofsky scale

## Abstract

**Purpose:**

This study aimed to investigate the association between clinical factors and temporary changes in functional performance in patients undergoing hemodialysis.

**Methods:**

This was a retrospective, longitudinal observational study conducted from 2015 to 2017. Eight-two patients undergoing hemodialysis in the outpatient clinic were enrolled. Functional performance was measured using the Karnofsky Performance Status (KPS) scale. Collected data for analysis included demographics, laboratory parameters, and KPS scale scores. All participants were grouped into a high KPS cluster and a low KPS cluster based on dynamic changes in KPS scales from 2015 to 2017.

**Results:**

Participants in the high KPS cluster demonstrated an approximate trend, and those in the low KPS cluster demonstrated a low pattern. By stepwise selection model analysis, age (OR 1.12, 95% CI 1.03–1.23, *p* = 0.011), serum BUN (OR 1.08, 95% CI 1.02–1.16, *p* = 0.015), calcium levels (OR 3.24, 95% CI 1.2–8.73, *p* = 0.02), and beta-2-microglobulin (OR > 1.0, CI >1.00-<1.01, *p* = 0.031) showed risk for the low KPS cluster. Male sex (OR 0.20, 95% CI 0.04–0.96, *p* = 0.045) and albumin level (OR 0.02, 95% CI 0–0.4, *p* = 0.009) showed a low risk for the low KPS cluster.

**Conclusions:**

A different trajectory pattern was observed between the high and low KPS clusters in a 3-year period. Risk factors for the low KPS cluster were age, serum BUN, calcium, and beta-2-microglobulin levels. Male sex and serum albumin levels reduced the risk for the low KPS cluster.

## Introduction

The association between functional performance status and chronic kidney disease (CKD) has been recognized [1–9]. CKD would elicit several physiological alterations and results in mineral bone disease, chronic inflammation, and vascular disease [10–13]. These alternations can lead to sarcopenia and weakness, both of which are the main realms of poor functional performance. Various clinical factors have been associated with functional performance decline in CKD including ethnicity, age, nutritional status, comorbidities, inflammation, hemodialysis (HD) frequency, and dialysis adequacy [6,8,9]. The complexity of these associations with functional performance can lead to detrimental clinical outcomes, specifically in the elderly population [1,8,14,15].

For nephrologists, the recognition of functional performance is important as the majority of patients with CKD are aged, and it has an impact on the decision of clinical management. In Taiwan, the Taiwan Society of Nephrology (www.tsn.org.tw) has applied a simple measurement of functional performance status in patients undergoing HD for years [i.e. the Karnofsky Performance Status (KPS) scale]. Initially, the KPS scale has been widely used to quantify the functional status of patients with cancer [16]. A modified version of the KPS scale was later proposed owing to the noted pitfalls and applied to study the functional status of patients undergoing HD [1]. It comprises 14 different levels of activity, ranging from <30 (hospitalized, progressive disability process) to ≥96 (normal function). In our previous studies, we have reported on the association between the KPS scale scores and clinical factors and its impact on the outcomes of patients undergoing HD [8,9].

Although age and chronic disease are the main determinants of functional performance status in CKD, there also exist some reversible conditions that can be managed or modified to improve functional performance, such as nutritional status, dialysis prescriptions, and physical endurance. A trajectory of one component of functional performance, namely, frailty, has been examined in a study over a 2-year period. The investigators found approximately equal numbers of patients with improving and worsening frailty scores over 2 years. The main associated factors for worsening frailty are markers of inflammation and hospitalization [6]. Considering that there are no studies that reported the temporary changes in KPS scale scores in patients undergoing HD, we aimed to investigate the association between clinical factors and trajectory of KPS scales in patients undergoing HD over a 3-year period.

## Materials and methods

### Participants

Patients who underwent regular outpatient HD at the Kaohsiung Chang Gung Memorial Hospital in Taiwan were screened for this study. The inclusion criteria were weekly maintenance HD and age above 18 years. The exclusion criteria were as follows: cardiac failure (left ventricular ejection fraction <50%), incomplete medical information, cancer history, and lost to follow-up during the study period. The included participants were followed up from January 1, 2015 to December 31, 2017. All participants underwent thrice weekly HD via the peripheral arteriovenous fistula and using dialyzers that had an effective surface area of >2.0 m^2^ and comprised cellulose acetate or polysulfone. HD duration was 4 h per session. The blood flow rate ranged from 280 to 300 cc/min, and dialysate calcium (Ca) and bicarbonate concentrations were 3.0 and 32 mEq/L, respectively.

The study protocol was approved by the Committee on Human Research at Kaohsiung Chang Gung Memorial Hospital (number: 104-2572B). Written informed consent was obtained from the participants. The study was conducted in accordance with the Declaration of Helsinki.

### Demographic, clinical, and laboratory variables

Data collection was performed for demographic information including age and sex. The clinical variables included a history of diabetes, primary kidney diseases, and HD vintage. Collected laboratory variables were hemoglobin (Hb), albumin, blood urea nitrogen (BUN), creatinine (Cr), corrected Ca, phosphate (P), potassium (K), intact parathyroid hormone (iPTH), p-cresyl sulfate (PCS), indoxyl sulfate (IS), beta-2-microglobulin, and fractional solute removal per dialysis treatment (Kt/*V*) urea. Kt/*V* urea was calculated using the following formula: –ln (*R* − 0.008 × *t*) + (4 – [3.5 × *R*]) × UF/W, where *R* is the ratio of post-dialysis BUN to pre-dialysis BUN, *t* (in minutes) is the duration of dialysis, UF (L) is the ultrafiltrate amount, and W (kg) is the post-dialysis body weight. Hb, albumin, BUN, Cr, corrected Ca, P, and K levels were measured once per month, and mean values obtained in 2015 were the baseline. iPTH, PCS, IS, and beta-2-microglobulin levels were measured at the beginning of the study in 2015. Laboratory values for blood analysis were measured midweek (on Wednesday or Thursday) *via* a venous port prior to HD following overnight fasting. All blood samples were tested using commercial kits and an autoanalyzer (Hitachi 7600-210, Hitachi Ltd., Tokyo, Japan). The albumin level was measured using the bromocresol green method, while the iPTH level was measured using chemiluminescence immunoassay (Siemens Healthcare Diagnostics Inc., Malvern, PA). The PCS and IS were quantified by performing high-performance liquid chromatography (Agilent 1100 Series, USA). Beta-2-microglobulin level was measured by performing turbidimetry (SPAPLUS, The Binding Site Group Ltd, UK).

### Karnofsky performance status scale

KPS scale scores were recorded by trained nurses at the HD unit upon enrollment of the participants. The record process by HD nurses included in-person observation in HD unit and interview with patients and their caregivers. The functional domain in KPS scoring was physical performance. The KPS scoring system comprises 14 different levels of activity, ranging from <30 (hospitalized, progressive fatal process) to ≥96 (normal function, no disability) [1]. The KPS scale score was obtained in December of each year from 2015 to 2017.

### K-means clustering

We used *k*-means clustering algorithm to divide 3-year KPS scale scores into two clusters (high and low KPS clusters) in which each observation belongs to the cluster with the nearest mean. The *k*-means starts with two cluster centers that are chosen at random initially. Then, the algorithm obtains a few iterations to recalculate the cluster center and assigns the samples to its nearest cluster center. This process is repeated until a convergence criterion is met. The characteristic of *k*-means clustering algorithm allowed us to dichotomize the patients into two clusters, taking into consideration the 3-year KPS scale score simultaneously. The three-dimensional *k*-means clustering plot was generated using Python’s “Matplotlib” (Hunter, 2007, URL https://ieeexplore.ieee.org/document/4160265/).

### Statistical analyses

Independent two-sample *t*-test, chi-squared test, or Fisher’s exact test were used to determine the different baseline characteristics between the high and low KPS clusters. Univariate and multivariate logistic regression analyses were used to demonstrate the association between baseline characteristics and KPS cluster. We revealed two multivariate models, including the fully adjusted and stepwise multivariate selection models, to clarify the comprehensive association between baseline characteristics and KPS cluster. The fully adjusted model considered all baseline characteristics as covariates. In the stepwise selection model, only the baseline characteristics with a *p*-value < 0.2 in the univariate analysis were selected as covariates. A *p*-value < 0.05 was considered statistically significant. All statistical analyses were performed using Stata (StataCorp. 2009. Stata 11 Base Reference Manual. College Station, TX: Stata Press).

## Results

**Figure 1.**
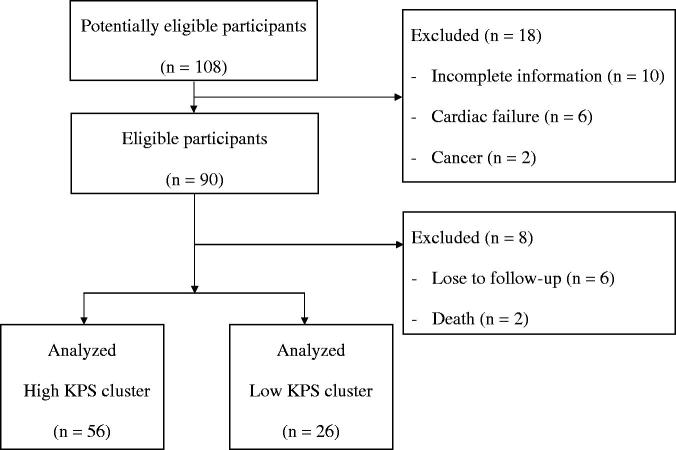
Participants’ flow diagram.

**Figure 2.**
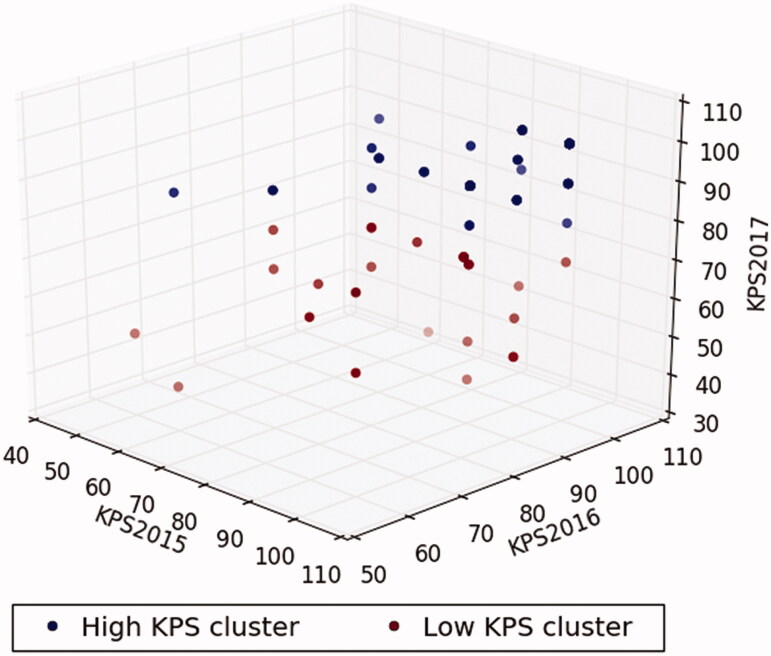
Three-dimensional plot using the Karnofsky Performance Status scores from 2015 to 2017 based on K-means clustering algorithm. High KPS cluster, navy dots; low KPS cluster, maroon dots.

A total of 82 participants were selected after the initial screening and complete observation in the study period (Figure 1). All participants were grouped into the high and low KPS clusters using *k*-means clustering algorithm in consideration of the dynamic change in KPS scale scores from 2015 to 2017. The numbers of patients in the high and low KPS clusters were 56 and 26, respectively. The scatter plot is shown in Figure 2. The two clusters were visibly separated. Participants in the high KPS cluster demonstrated an approximate trend. In contrast, participants in the low KPS cluster demonstrated a tendency toward the low KPS cluster during the study period.

The baseline characteristics of participants are shown in Table 1. Participants with low KPS cluster were older than those with high KPS cluster. Comparing the laboratory parameters, serum albumin level was significantly higher in participants in the KPS cluster than that in participants in the low KPS cluster. The other laboratory parameters, including biochemical variables and Hb, Kt/V, iPTH, PCS, IS, and beta-2-microglobulin levels, were not significantly different between the two clusters.

**Table 1.** Baseline characteristics of hemodialysis patients categorized by KPS cluster (derived from KPS scores between 2015 and 2016) (*N* = 82).

Variables	High KPS cluster (*n* = 56)	Low KPS cluster (*n* = 26)	*p*
Age (years)	61 ± 11	67 ± 7	0.008
Gender (male, %)	25 (44.64%)	10 (38.46%)	0.598
Dialysis vintage (years)	11.0 ± 6.90	10.0 ± 5.96	0.516
Diabetes	10 (17.86%)	9 (34.62%)	0.158
Antihypertensive drugs	15 (26.78%)	8 (30.77%)	0.709
Primary kidney disease			0.547
Chronic glomerulonephritis	14 (25.00%)	4 (15.38%)	
Hypertensive nephropathy	25 (44.64%)	16 (61.54%)	
Chronic interstitial nephritis	1 (1.79%)	—	
Cause unknown	16 (28.57%)	6 (23.08%)	
KPS scale			
2015	90.54 ± 10.52	86.92 ± 13.79	0.195
2016	92.50 ± 8.37	80.38 ± 13.11	<0.001
2017	93.39 ± 5.81	65.38 ± 13.92	<0.001
Laboratory data			
Hb (g/dL)	10.71 ± 1.31	10.52 ± 0.94	0.51
Albumin (g/dL)	3.99 ± 0.28	3.77 ± 0.31	0.002
BUN (mg/dL)	61.29 ± 0.28	63.73 ± 15.65	0.545
Cr (mg/dL)	10.08 ± 2.45	9.64 ± 1.77	0.413
Ca (mg/dL)	9.34 ± 1.05	9.60 ± 0.86	0.272
P (mg/dL)	6.66 ± 11.83	4.82 ± 1.43	0.433
K (meq/L)	4.58 ± 0.74	6.17 ± 9.98	0.236
Kt/V	1.83 ± 0.40	1.72 ± 0.23	0.214
iPTH^a^ (pg/ml)	205.05 (68.80–433.35)	293.7 (108.80–664.90)	0.259
PCS(ug/ml)	27.09 ± 20.30	30.59 ± 27.05	0.516
IS (ug/ml)	43.53 ± 23.50	40.09 ± 17.05	0.507
Beta-2-microglobulin^a^ (ug/L)	25918.80 (20300.00–30199.60)	28240.70 (20241.80–35610.00)	0.213

BUN: blood urea nitrogen; Cr: creatinine; Ca: calcium; Hb: hemoglobin; iPTH: intact parathyroid hormone; IS: indoxyl sulfate; K: potassium; KPS: Karnofsky performance status; P: phosphate; PCS: p-cresyl sulfate.

^a^Median, interquartile range.

*p*-value was estimated using two-sample *t*-test, chi-squared test or Fisher's exact test appropriately.

Based on logistic regression analysis with the high KPS cluster as a reference, age (odds ratio [OR] 1.15, 95% confidence interval [CI] 1.04–1.28, *p* = 0.008) and serum BUN level (OR 1.09, 95% CI 1.01–1.16, *p* = 0.022) in the fully adjusted model exhibited risk for the low KPS cluster. Based on stepwise selection model analysis, age (OR 1.12, 95% CI 1.03–1.23, *p* = 0.011) and serum BUN (OR 1.08, 95% CI 1.02–1.16, *p* = 0.015) and Ca levels (OR 3.24, 95% CI 1.2–8.73, *p* = 0.02) showed risk for the low KPS cluster. Male sex (OR 0.20, 95% CI 0.04–0.96, *p* = 0.045) and serum albumin level (OR 0.02, 95% CI 0–0.4, *p* = 0.009) showed a low risk for the low KPS cluster. Beta-2-microglobulin level revealed a high risk for the low KPS cluster by either the fully adjusted or stepwise selection model (Table 2).

**Table 2.** Logistic regression for KPS cluster (derived from KPS scores between 2015 and 2017) (*N* = 82)

Variables	Univariate			Full-adjusted model			Stepwise selection (*p* < 0.2) model		
	OR	95% CI	*p*	OR	95% CI	*p*	OR	95% CI	*p*
Dependent variables (reference: high KPS cluster)									
Age	1.07	1.02–1.13	0.012	1.15	1.04–1.28	0.008	1.12	1.03–1.23	0.011
Gender (male, %)	0.78	0.30–2.00	0.599	0.15	0.02–0.98	0.048	0.20	0.04–0.96	0.045
Dialysis vintage	0.98	0.91–1.05	0.511	0.92	0.80–1.06	0.265	0.89	0.79–1.01	0.066
Diabetes	2.44	0.84–7.02	0.099	1.91	0.34–10.83	0.464	—		
Hb	0.87	0.58–1.30	0.505	0.77	0.35–1.70	0.513	—		
Albumin	0.06	0.01–0.44	0.005	0.07	<0.01–1.45	0.086	0.02	<0.01–0.40	0.009
BUN	1.01	0.98–1.04	0.540	1.09	1.01–1.16	0.022	1.08	1.02–1.16	0.015
Cr	0.91	0.72–1.14	0.410	0.79	0.50–1.26	0.323	0.69	0.45–1.07	0.096
Ca	1.33	0.80–2.23	0.270	2.01	0.65–6.19	0.226	3.24	1.20–8.73	0.020
P	0.86	0.60–1.24	0.420	0.51	0.23–1.10	0.084	0.54	0.28–1.04	0.065
K	1.05	0.94–1.18	0.358	1.08	0.94–1.24	0.297	1.11	0.97–1.27	0.148
Kt/V	0.41	0.10–1.67	0.213	0.22	0.01–3.95	0.302	—		
iPTH	>1.00	<1.00–>1.00	0.260	>1.00	<1.00–>1.00	0.339	—		
PCS	1.01	0.99–1.03	0.513	1.01	0.98–1.05	0.479	—		
IS	>0.99	0.97–1.01	0.502	0.97	0.92–1.01	0.160	—		
Beta-2-microglobulin	>1.00	<1.00–>1.00	0.212	>1.00	>1.00–<1.01	0.047	>1.00	>1.00–<1.01	0.031

## Discussion

In this study, we examined the association between clinical factors and KPS scale score changes in patients undergoing HD for 3 years. We found that age and serum BUN, Ca, and beta-2-microglobulin levels were the risk factors for low KPS cluster. In contrast, male sex and serum albumin level were the positive factors for high KPS cluster. We also observed that patients with low KPS cluster in the study period revealed a tendency toward the low KPS cluster. Meanwhile, patients in the high KPS cluster maintained a constant tendency during the study period. These results revealed that there existed modified and non-modified clinical factors for KPS improvement in patients undergoing HD. We supposed that modified factors, including serum albumin, BUN, Ca, and beta-2-microglobulin levels could be improved by dietary education, enhanced HD clearance, and prescription review. Non-modified factors, such as age and sex, cannot be generally changed; however, some clinical practices, such as programmed rehabilitation, psychological evaluation, and drug modification, may reduce the impact of these factors on physical performance in patients undergoing HD.

One of the findings of our study is that the serum Ca level increases the risk for low KPS cluster over a 3-year period. At baseline, serum Ca level (mean values from 2015 to 2016) was higher in the low KPS cluster compared to that in the high KPS cluster (*p* = 0.272). This result is consistent with those in our previous reports [8,9]. The definitive causes for relatively high serum Ca level in the low KPS cluster cannot be drawn in our study owing to the incomplete history of Ca-containing pill and vitamin D analog intake, hypercalcemia-related diseases, and hypercalcemic dietary habits. Hypercalcemia indicated an increased risk of all-cause mortality in our HD cohort [17]. We hypothesized that there existed unidentified clinical factors in those with hypercalcemia in the low KPS cluster, which may lead to lower functional performance and higher death risk. However, the contribution of serum Ca level to functional performance in the population undergoing HD needs to be examined with a large-scale population study in the future.

We examined the traditional correlates of functional performance such as age, sex, and diabetes, which were associated with functional performance as expected. We found that old age was significantly associated with decreasing KPS scale scores over a 3-year period. This result is consistent with those of previous studies [3,9]. Moreover, our study revealed that the male sex presented a significantly reduced risk of lower KPS cluster in the observational period. Women were reported to have lower functional performance than men in not only the cross-sectional but also longitudinal study [6,9]. The detailed information on substantial difference in sex was not reported in previous studies. Nevertheless, the sex effect might be overcome by a sophisticated therapeutic plan and teamwork. This is the intrinsic value to echo the objective in our study.

It is well known that age and serum albumin levels are two components related to functional performance in the population with CKD [3,6,8,9,15,18–21]. It is reasonably realized that age and low serum albumin level could lead to physical disability. Lower serum albumin level reflects lower muscle mass and sarcopenia and, hence, reduced functional performance. Nevertheless, serum albumin level can be improved by exploring underlying clinical conditions. In clinical practice, a multidisciplinary team engaged in discussion and exploration can be expected to improve nutritional status in patients undergoing HD. We also believe that a trajectory of KPS scales in patients undergoing HD shows insight into the possible modified clinical factors to overcome poor functional performance.

We attempted to determine the association between uremic toxins and trajectory of functional performance in patients undergoing HD. We analyzed this association with the indicators of small uremic solutes and middle molecules in circulation. Our result did not exhibit a positive association between uremic toxins and trajectory of KPS scales in patients undergoing HD, except with BUN and beta-2-microglobulin. In our previous study, we found that BUN was one of the major determinants of functional performance in patients undergoing HD by the classification and regression tree approach [8]. It is well known that HD patients have high beta-2-microglobulin levels [22]. The deposit of beta-2-microglobulin is mainly in musculoskeletal system. The preferential deposition in tendons and bones can result in physical functional impairment [22–24]. Our findings elicit a hypothesized strategy to utilize large-pore hemodialyzers to remove large-size uremic toxins. The effects of improving physical functional impairment by these hemodialyzers in HD patients warrant to be investigated in the future. Our study also implied that holistic evaluation should be taken into account in making decisions for the management of impaired functional performance in patients undergoing HD. It is concerned with not only dialysis adequacy but also other potential contributors in clinical scenario.

In this study, patients with high KPS scores at baseline demonstrated a constant pattern during the study period. In contrast, patients with low KPS scores at baseline revealed a decline pattern. The possible explanations for low KPS cluster are older age, more prevalent with diabetes and lower serum albumin levels after abstracting from our dataset. The contribution of age in KPS pattern is consistent with a prior study [9]. Nevertheless, we suppose that the mutual interaction with various clinical factors could be the main determinant for low KPS cluster in our cohort. Based on our statistical analysis, we propose that age, sex, serum albumin, Ca and beta-2-microglobulin levels are these determinants. From this observation, we admit that non-modified demographic factors are difficult to overcome. Therefore, improving the functional physical performance of patients with low KPS cluster is considered difficult in the study period. A challenging issue elicits further study in the future.

The strength of our study included the demonstration of a trajectory of physical performance status and their associations with various demographic and clinical factors in patients undergoing HD. This study has the following limitations. This study included a relatively small sample size and nonrandomized selection of patients. Comorbidity and inflammation that may influence the physical performance of patients undergoing HD were also not included in the examination. Finally, functional status varies with acute illness, and over time, these changes can be relatively rapid over months with both patterns of improvement and decline. A longer more frequent evaluation system is required to understand trends.

## Conclusions

There is a tendency to improve the functional performance of patients undergoing HD in a 3-year observation period. The trend indicated a trajectory of KPS cluster with approximate trend in either the high or low KPS cluster. The main associated factors are age, male sex, and serum albumin, BUN, and Ca levels. Male sex and serum albumin level presented a reduced risk for low KPS cluster in a 3-year observation period. Conversely, age and serum BUN, beta-2-microglobulin, Ca levels presented a risk for low KPS cluster. Accordingly, our study implicates that the main modified factors for high KPS scores in HD patients are nutritional status and serum Ca levels. Moreover, adequate HD especially in large-size uremic toxins is also essential for maintaining high KPS scores in HD patients. A sophisticated strategy to handle dynamic changes in clinical parameters is critical in clinical practice. Future studies should examine whether interventions through multidisciplinary teamwork could overcome these clinical factors and improve functional performance in patients undergoing HD.

## Data Availability

The raw data used to support the findings of this study are available from the corresponding author upon reasonable request.
